# Combining Transient Expression and Cryo-EM to Obtain High-Resolution Structures of Luteovirid Particles

**DOI:** 10.1016/j.str.2019.09.010

**Published:** 2019-12-03

**Authors:** Matthew J. Byrne, John F.C. Steele, Emma L. Hesketh, Miriam Walden, Rebecca F. Thompson, George P. Lomonossoff, Neil A. Ranson

**Affiliations:** 1 Astbury Centre for Structural Molecular Biology, School of Molecular & Cellular Biology, Faculty of Biological Sciences, University of Leeds, Leeds LS2 9JT, UK; 2 Department of Biological Chemistry, John Innes Centre, Norwich Research Park, Colney, Norwich NR4 7UH, UK

**Keywords:** cryo-EM, homology modeling, virus, plant virus, structure, luteovirus

## Abstract

The Luteoviridae are pathogenic plant viruses responsible for significant crop losses worldwide. They infect a wide range of food crops, including cereals, legumes, cucurbits, sugar beet, sugarcane, and potato and, as such, are a major threat to global food security. Viral replication is strictly limited to the plant vasculature, and this phloem limitation, coupled with the need for aphid transmission of virus particles, has made it difficult to generate virus in the quantities needed for high-resolution structural studies. Here, we exploit recent advances in heterologous expression in plants to produce sufficient quantities of virus-like particles for structural studies. We have determined their structures to high resolution by cryoelectron microscopy, providing the molecular-level insight required to rationally interrogate luteovirid capsid formation and aphid transmission, thereby providing a platform for the development of preventive agrochemicals for this important family of plant viruses.

## Highlights

• Establish method for producing high-titer luteovirus VLPs in plants
• Presents high-resolution cryo-EM structure of BYDV and PLRV VLPs
• Provides platform for rational interrogation of luteovirid capsid formation
• Coat protein models for the type-species in each of the three Luteoviridae genera

## Graphical Abstract

**Figure:**
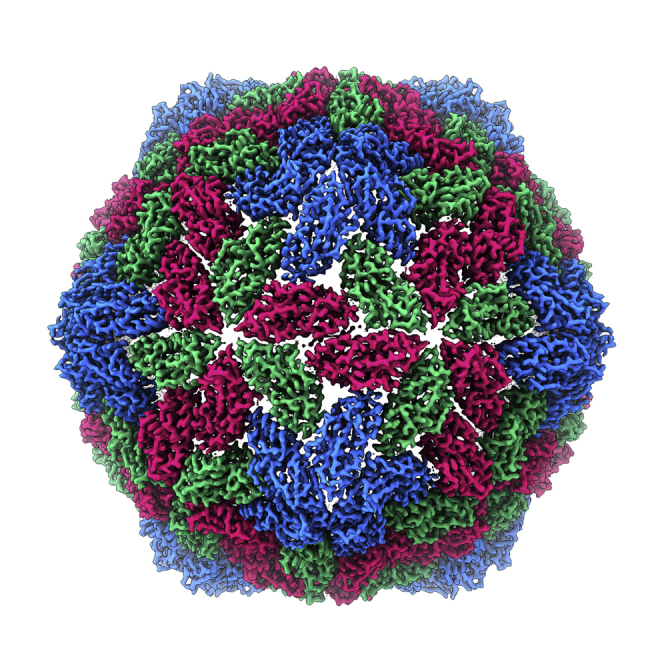

## Teaser

The Luteoviridae include viruses that cause enormous crop losses worldwide. Byrne et al. present high-resolution cryo-EM structures of barley yellow dwarf and potato leafroll viruses, revealing the structure of the luteovirus capsid and providing a platform to interrogate capsid formation and aphid vectored transmission of this important family of viruses.

## Introduction

Plant virus infection is responsible for global economic losses estimated at >$30 billion each year (International Committee on Taxonomy of Viruses, 2012). The Luteoviridae are an important family of pathogenic viruses responsible for necrosis of the plant vasculature which, in turn, causes severe stunting and dwarfism, and ultimately crop loss. The Luteoviridae contains some of the most damaging crop pathogens, including barley yellow dwarf virus (BYDV) and potato leafroll virus (PLRV), which cause crop losses to a value of £40–60 million annually in the United Kingdom alone (Wale et al., 2008). The family is subdivided into three genera, the luteoviruses, the poleroviruses, and the enamoviruses, referred to collectively as the luteovirids.

**Figure 1:**
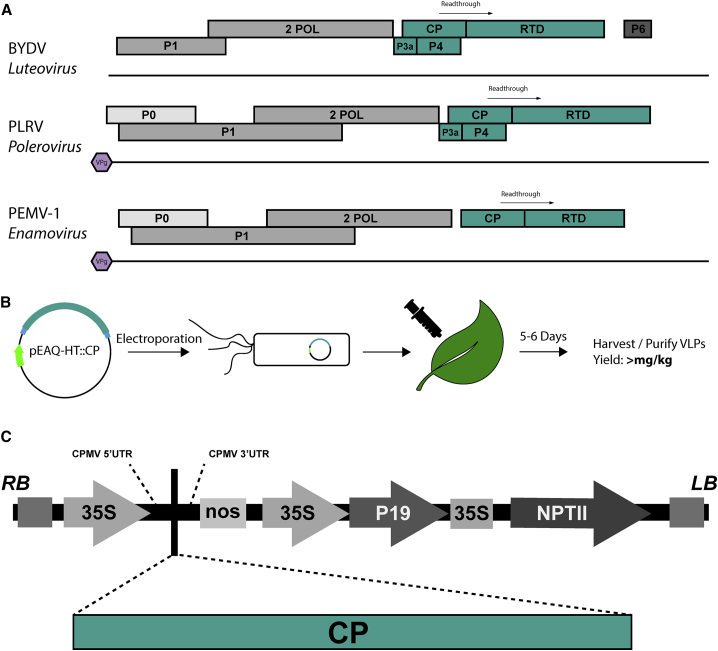
Transient Expression of luteovirid VLPs in Plants (A) Schematic representation of genomes from each of the Luteoviridae genera. Genes colored in teal comprise the “luteovirid block.” (B and C) Scheme illustrating pEAQ-*HT* transient expression of luteovirid VLPs (B) and detailed schematic of the pEAQ-HT vector, highlighting important genetic elements (C). Within the pEAQ-*HT* vector, the cauliflower mosaic virus (CaMV) 35 promoters are indicated by arrows while the nopaline synthase (nos) and CaMV 35S terminators are indicated by boxes. The 5′ and 3′ CPMV untranslated regions (UTRs) between which the sequences of the coat proteins are inserted are indicated. RB and LB represent the left and right transfer DNA borders, respectively, P19 encodes the P19 suppressor of gene silencing, and NPTII encodes neomycin phosphotransferase II, which confers kanamycin resistance.

Luteovirids have a single-stranded positive sense RNA genome ((+)ssRNA), ranging between 5.3 and 6.0 kb in length, which encodes five or six open reading frames (ORFs), designated ORF 0–6 (Figure 1). Poleroviruses encode protein ORFs 0–5, while luteoviruses encode ORFs 1–5, and enamoviruses ORFs 0–3 and 5–6. ORF0 encodes a suppressor of viral RNA silencing. ORF1 and ORF2 encode the P1 and P2 elements of the viral RNA-dependent RNA polymerase (RdRp). ORF3 encodes the coat protein (CP). ORF4 encodes a movement protein, required for cell-to-cell movement of the viral RNA. ORF5 encodes a readthrough domain, which is incorporated into a subset of CPs by way of a readthrough of the ORF3 stop codon, to yield the so-called coat protein-readthrough domain (CP-RTD). Additionally, polerovirus and luteovirus RNAs contain an additional ORF, termed ORF3a, upstream of ORF3 that starts with a non-AUG codon and overlaps ORF3; the product of this is involved in long-distance movement of the virus (Smirnova et al., 2015, DeBlasio et al., 2018). Enamoviruses, including the type member, pea enation mosaic virus 1 (PEMV1), lack a movement protein (ordinarily P4), which is instead provided *in trans* by an obligatory co-virus, the umbravirus PEMV2, which can replicate independently (Demler et al., 1993). The presence of PEMV2 allows PEMV1 to move out of the phloem and can also potentiate the movement of other luteovirids (Ryabov et al., 2001). Although ORF0 is not present in luteoviruses, ORF4 is thought to provide suppression of RNA silencing in addition to acting as the movement protein (Fusaro et al., 2017).

Transmission of this family of viruses is facilitated by aphid vectors in a circulative, non-propagative manner. The virus is taken up through an aphid's feeding apparatus into its gut, where it is transcytosed into the hemocoel (Linz et al., 2015). Virus is then circulated through the body cavity of the aphid in the hemolymph and binds to receptors on the accessory salivary gland, where it is again transcytosed and suspended in the insect's saliva, before being deposited in the phloem of plants upon feeding (Miller et al., 2002). Viral propagation is limited to the phloem and no replication occurs in the insect vector. ORFs 3–5 form the so-called luteovirid block (Figure 1), which is conserved across all luteovirids, and is ultimately responsible for the signature luteovirid phloem-specific tropism (ORF4/ORF5) and aphid vector range specificity (CP/CP-RTD) (Miller et al., 2002, Brault et al., 2005, Peter et al., 2009).

Luteovirid capsid structures and the interactions they make with aphid vectors during transmission have been of interest to researchers for many years (Gray and Gildow, 2003). However, to date these studies have been limited to computational modeling and biochemical/biophysical interrogation of capsid proteins (Torrance, 1992, Terradot et al., 2001, Brault et al., 2003, Kaplan et al., 2007, Chavez et al., 2012, Alexander et al., 2017). This is, in large part, the result of difficulties isolating sufficient luteovirid virions for analysis. The phloem limitation of most luteovirids results in a low viral titer in wild-type infections, even in the laboratory where infection can be targeted and controlled. This has hindered the elucidation of high-resolution luteovirid capsid structures, owing to the relatively large (>1 mg) quantities of purified virus required for X-ray crystallography.

There have been several attempts to circumvent the problem of low viral titers associated with infections. Hoffmann et al. (2001) and Yoon et al. (2011) used ballistic bombardment and agroinfiltration, respectively, to infect whole plants with luteovirids. However, in neither case was any attempt made to purify virus particles. The CPs of luteoviruses beet western yellows virus and PLRV have also been expressed in insect cells or plants (Tian et al., 1995, Lamb et al., 1996, Skurat et al., 2017). The expressed proteins were able to form virus-like particles (VLPs), but no structural studies were performed.

We have previously shown that plant-based transient expression using the pEAQ-*HT* vector system yields VLPs that are accurate immunological and structural mimics of the authentic virus (Sainsbury et al., 2009, Peyret and Lomonossoff, 2013, Hesketh et al., 2015, Hesketh et al., 2017, Marsian et al., 2017). Here, we have utilized this approach for the recombinant expression of luteovirid VLPs in plants (Figure 1), and have used cryoelectron microscopy (cryo-EM) to overcome the barriers that have stood in the way of structural characterization of luteovirid capsids for many years. We therefore present high-resolution structures for VLPs of BYDV and PLRV at 3.0-Å and 3.4-Å resolution, respectively. The third type of luteovirid species, PEMV1, proved recalcitrant to forming ordered VLPs, an issue previously encountered during expression of the PEMV1 CP in insect cells (Sivakumar et al., 2009). We were not, therefore, able to determine its cryo-EM structure-, but given the high sequence homology between BYDV, PLRV, and PEMV1, we used homology modeling, and the new BYDV and PLRV structures to generate a structure for PEMV1, providing complete structural coverage for viruses in the Luteoviridae.

## Results and Discussion

### Expression and Characterization of Luteovirid Particles

**Figure 2:**
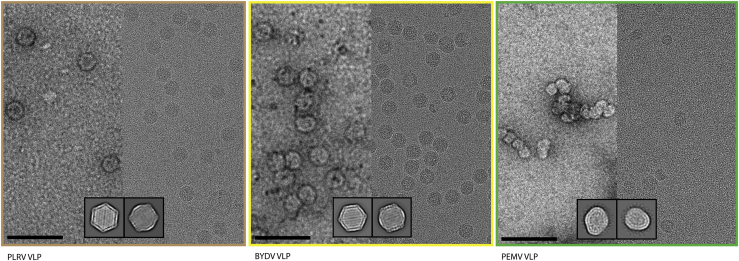
BYDV and PLRV VLPs Form Stable Capsids Representative cryo-EM and negative-stain micrographs for each of the luteovirid VLPs and PEMV WT virions. Inset: representative 2d classes from cryo-EM data collection. (Left) Potato leafroll virus VLPs. (Middle) Barley yellow dwarf virus VLPs. (Right) Pea enation mosaic virus 1 VLPs (see also Figure S4). Scale bar represents 100 nm at magnifications shown.

To allow structural characterization of the luteovirid type-species capsids by cryo-EM, we expressed BYDV, PLRV, and PEMV VLPs in *Nicotiana benthamiana* using the pEAQ-*HT* vector system. This system allows the placement of the sequence to be expressed between a modified 5′ untranslated region (UTR) and the 3′ UTR from cowpea mosaic virus RNA-2, ensuring high levels of expression of the resulting mRNA. Expression is further enhanced by co-expression of the P19 suppressor of gene silencing (Sainsbury et al., 2009, Peyret and Lomonossoff, 2013). The system is particularly effective for the production of VLPs in plants (Thuenemann et al., 2013, Marsian and Lomonossoff, 2016). The CP sequences were codon-optimized for expression in *N*. *benthamiana*, which disrupted ORF4 in the case of PLRV and BYDV; ORF3A was not included in the inserted sequences. The pEAQ-*HT* plasmids containing the genes encoding the appropriate luteovirid CP were transformed into *Agrobacterium tumefaciens* and used to agroinfect *N*. *benthamiana*. VLPs were purified from infiltrated leaves and analyzed by negative-stain electron microscopy (Figure 2). BYDV and PLRV CPs assembled into monodisperse, homogeneous particles of the anticipated diameter (Figure 2). However, PEMV1 CP assembled into amorphous, aggregation-prone particles, a phenomenon that persisted when imaged using cryo-EM (Figure 2). This result agrees with those observed for baculovirus-derived PEMV VLPs (Sivakumar et al., 2009). Despite extensive screening of buffer conditions during purification and grid preparation (not shown), we could not remedy the observed heterogeneity for PEMV1.

**Figure 3:**
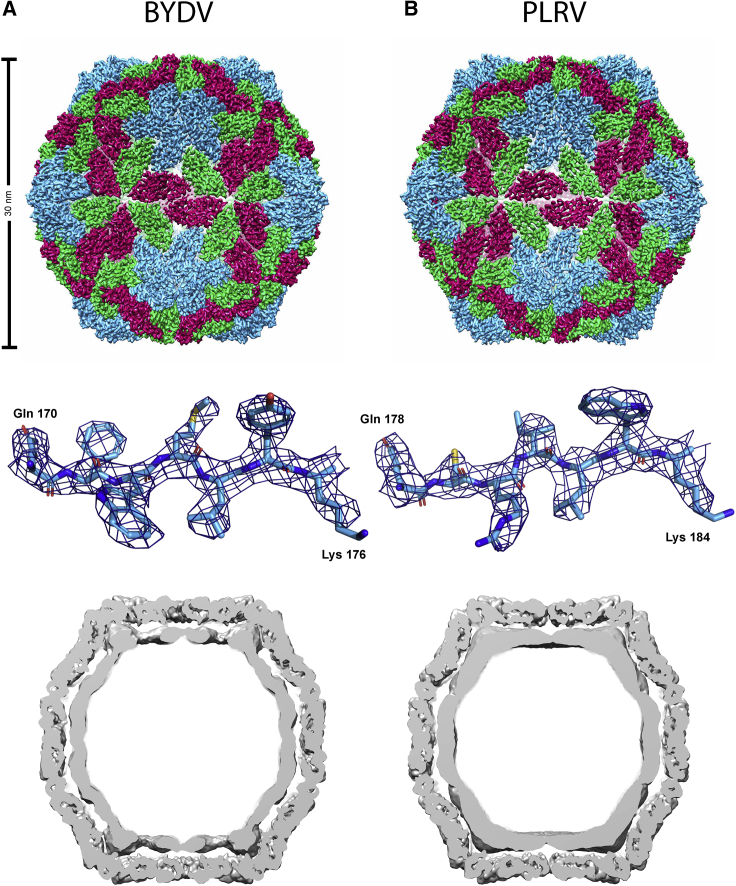
Cryo-EM Structures of Luteovirid Capsids BYDV (A) and PLRV (B). Top: cryo-EM maps of whole virus capsid, colored according to CP quasi-conformers, where subunit A is blue, subunit B is green, and subunit C is red. Middle: section of representative density and molecular model for each virus. Bottom: slice through unsharpened maps, depicting density for packaged RNA and/or disordered R domain (see also Figure S5).

When imaged in ice, both BYDV and PLRV VLPs were monodisperse (Figure 2), so cryo-EM datasets were collected for each (see STAR Methods). Structure refinement was carried out with icosahedral symmetry imposed, yielding density maps at a resolution of 3.0 Å for BYDV and 3.4 Å for PLRV (Figure 3; see Table S1 for refinement statistics). The primary sequences of the relevant viral CPs were used to generate initial models by automated homology modeling. Final models were then built as a single asymmetric unit, comprising three chains (A, B, and C) (Figure 3).

The structures for BYDV and PLRV each reveal a luteovirid capsid composed of 180 CP monomers. The CP monomers each contain a single canonical jellyroll fold, and are arranged with *T* = 3 icosahedral quasi-symmetry to give a particle with a diameter of ∼30 nm. Density maps for each structure were of sufficient quality and resolution to allow for unambiguous building of the shell (S) domain of the CP (BYDV: residues 61–200^∗^; PLRV: residues 68–208). In both cases the N-terminal R (RNA-binding) domain is not resolved in the cryo-EM density, except in the “C” conformer of BYDV, where an additional six residues of the R domain (residues 55–60) are visible. Many plant virus structures, e.g., STNV, contain metal ions that stabilize the capsid, and play important roles in transmission and infectivity. No density for such metal ions is seen in either BYDV or PLRV, and no putative metal binding sites were identified using bioinformatics. As for many viruses, both capsid structures are strongly positively charged on their inner surfaces (Figure S1), presumably to help facilitate packaging of the polyanionic RNA genome. The outer surface charge profile is similar in both BYDV and PLRV capsid structures, with an acidic patch at the center of each asymmetric trimer (Figure S1). Amorphous density is found within each particle structure, presumably representing packaged RNA and/or R domains that are not ordered with icosahedral symmetry (Figure 3). There is no ordered density within either structure. This agrees with previous observations that luteovirid VLPs are capable of non-specifically packaging RNAs from the expression host (Sivakumar et al., 2009). Although we have not specifically analyzed the RNA content of the plant-produced luteovirid VLPs, we anticipate that they will contain plant-derived RNAs as has been reported for other plant-expressed VLPs (e.g., cucumber necrosis virus and satellite tobacco necrosis virus) (Ghoshal et al., 2015, Alam et al., 2017, Kotta-Loizou et al., 2019). Similar results are also seen with ssRNA virus VLPs recombinantly expressed in insect cells (Sivakumar et al., 2009, Routh et al., 2012). We do not anticipate that the presence of host-derived RNA will affect the architecture of the capsid. Inspection of the interfaces between individual CP protomers, and between asymmetric units, in BYDV and PLRV suggest that both viruses have a similar volume of solvent hidden surface involved in supramolecular assembly (Tables S4 and S5). We have also solved the structure of VLPs formed by an N-terminally His-tagged version of PLRV CP to 3.4-Å resolution, since the formation of His-tagged VLPs were previously reported (Lamb et al., 1996). No differences were found between the structures of the VLPs formed by tagged and untagged CPs.

### Homology Modeling of the PEMV1 Structure

**Figure 4:**
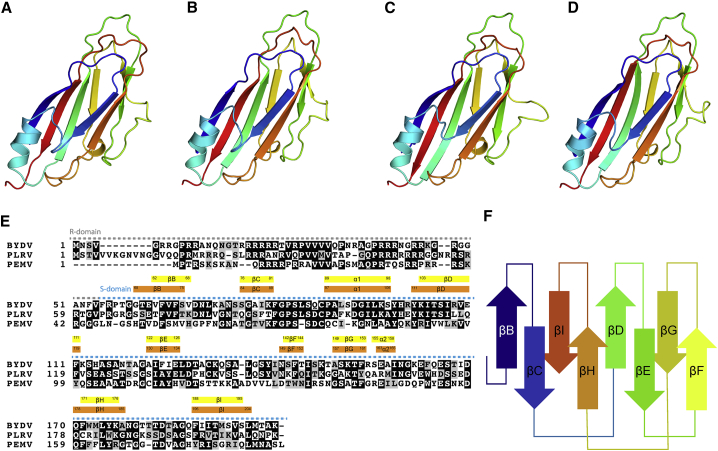
Comparison of Luteovirid Coat Protein Structures (A–D) BYDV (A), PLRV (B), PEMV^BYDV^ (C), and PEMV^PLRV^ (D), colored blue at the N terminus through to orange at the C terminus. (E) Sequence alignment of BYDV, PLRV, and PEMV. Labels above sequence indicate CP domains. Secondary structure elements are indicated by colored boxes. Yellow boxes denote BYDV secondary structure. Orange boxes denote PLRV secondary structure. (F) Schematic of the jellyroll fold, colored according to CP quasi-conformers (A–C). See also Figures S2, S3, and S7.

To gain an insight into the structure of PEMV and demonstrate the utility of our structural data, we generated homology models of PEMV using the SWISS-MODEL server (Schwede et al., 2003). Separate homology models were generated using our BYDV and PLRV structures as templates, termed PEMV^BYDV^ and PEMV^PLRV^, respectively. Superposition of the independently derived PEMV^BYDV^ and PEMV^PLRV^ structures yielded a root-mean-square deviation (RMSD) of 0.8 Å, comparable with the RMSD obtained when comparing BYDV and PLRV. The PEMV^BYDV^ and PEMV^PLRV^ models predict placement of core secondary structure elements with a high degree of structural identity (Figures 4C and 4D). Structural superposition of PEMV^BYDV^ and PEMV^PLRV^ excluding residues in loop regions yields an RMSD of 0.6 Å, while superposition of PEMV^BYDV^ and PEMV^PLRV^ excluding residues in β strands and α helices yields, as expected, a larger RMSD of 1.2 Å.

To test our hypothesis that BYDV and PLRV structures are likely to serve as good templates for homology modeling of luteovirid capsid structures for which there is currently no structural data (i.e., PEMV), we generated homology models of BYDV and PLRV using the other as a structural template, termed BYDV^PLRV^ and PLRV^BYDV^. Structural superposition of these models with their respective experimentally determined structures yielded RMSDs of 1.07 Å for BYDV and 0.84 Å for PLRV. Collectively these data show that the BYDV and PLRV structures presented herein, together with the sequence/structure conservation within the Luteoviridae, allow extremely accurate homology modeling of all luteovirid capsids.

### Comparison of Luteovirid Capsid Structures

**Figure 5:**
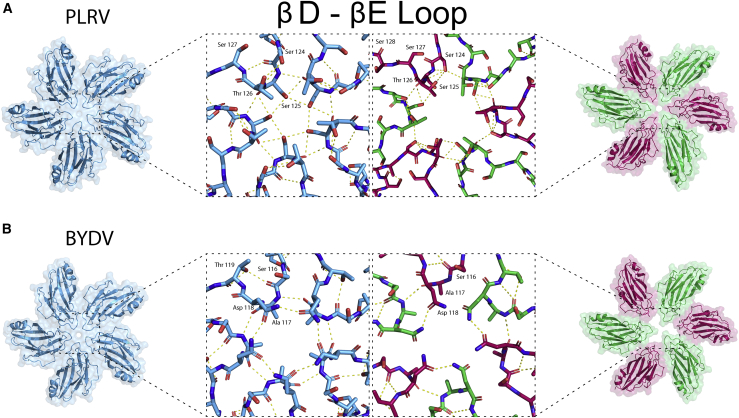
Structural differences at the annuli of PLRV and BYDV PLRV (A) and BYDV (B). Left: five-fold annuli. Inset: stick representation of residues depicting hydrogen-bonding network. Right: three-fold annuli. Inset: stick representation of residues depicting hydrogen-bonding network.

The luteovirid capsids are formed from CP subunits with a canonical jellyroll fold comprising two opposing β sheets, each containing 4 antiparallel β strands (βB-βI). This core structure is decorated with two helices (α1 and α2), with α1 situated between βC and βD, and α2 forming a short helical turn on the loop between βG and βH. Pairwise superposition of the backbones of BYDV, PLRV, and PEMV CPs in any combination yields an RMSD of <1 Å, demonstrating that the structural similarity across all three structures is high. When comparing subunits A–C from a single asymmetric unit within each virus, the Cα atoms superpose with almost complete structural identity, with the βD-βE loop showing the highest degree of structural variation. The subtly different conformations taken by the βD-βE loop, in all three capsid structures, are crucial in achieving the quasi-equivalent inter-chain interactions integral to a *T* = 3 icosahedron assembly (Figure 5).

### Luteovirids Are Structurally Similar to Picorna-like Viruses

The Luteoviridae are currently not assigned to a particular viral Order, although suggestions have been made to group them with a variety of plant viruses based upon genetic similarities (Miller et al., 2002). Elsewhere in the literature, Stuart and colleagues have made powerful arguments for grouping viruses based upon their structural similarity, allowing common ancestry to be inferred even when genetic similarity is no longer detectable (Abrescia et al., 2012). This structural conservation may be due to selection pressures that dictate a virus must retain absolute capsid integrity to allow propagation. To position the luteovirids in “viral structure space,” we carried out bioinformatics analysis of the BYDV and PLRV CP structures using the Dali server (Holm and Rosenström, 2010). Four distinct structural lineages have been previously established, and a Dali search, against the entire PDB, suggests that the luteovirids sit within the picorna-like lineage. Correspondence analysis of luteovirid CP structures against representative members from all known picorna-like families establish the Luteoviridae's nearest structural neighbors (Figure S2), and thus allow the construction of a structural dendrogram (Figure S3). The Luteoviridae bear significant structural similarity to members of four viral families that exclusively infect plants (the Secoviridae, Tymoviridae, Tombusviridae, and Solemoviridae), and a single family (the Astroviridae) with vertebrate hosts.

**Figure 6:**
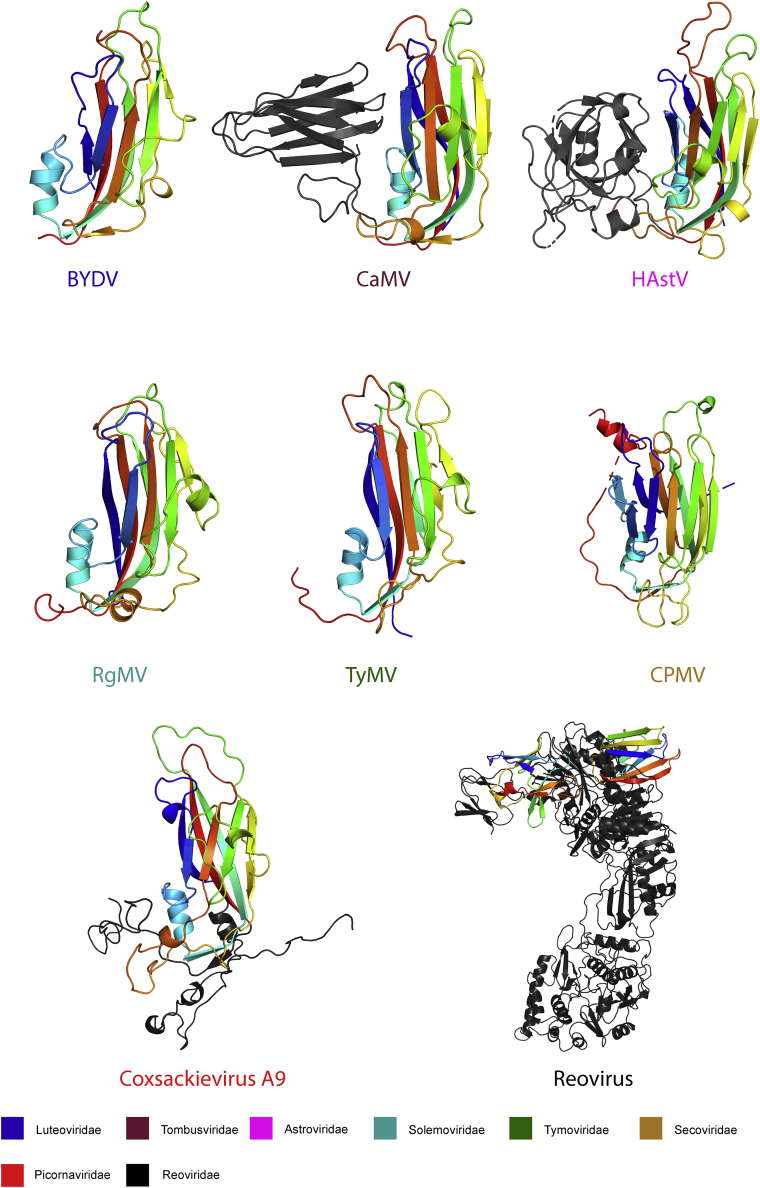
Comparison of the Luteovirid CP (BYDV) with Representative Members of Picorna-like Lineage Members A single member of the Pircornaviridae, and the structurally distinct BTV-like lineage, are included for comparison. Jellyroll fold is colored blue at the N terminus through to red at the C terminus for ease of comparison. Regions outside of the jellyroll are colored gray for clarity (see also Figures S2 and S3).

Structural superpositions of the BYDV and PLRV with members of these families shows that the luteovirid CP is undecorated, describing a prototypical jellyroll fold comprising eight β strands and two α helices. BYDV and PLRV have comparatively short insertions at the C-D, E-F, and G-H loops, where larger insertions are found in all other viruses with known structures (Figure 6), resulting in a much more compact fold (Khayat and Johnson, 2011). Prototypical helices on the C-D and G-H loops are retained in the luteovirid CPs, with the C-D helix (α1) comprising ten residues, as in all structures compared here, whereas the G-H (α2) helix forms a comparatively short helical turn.

**Figure 7:**
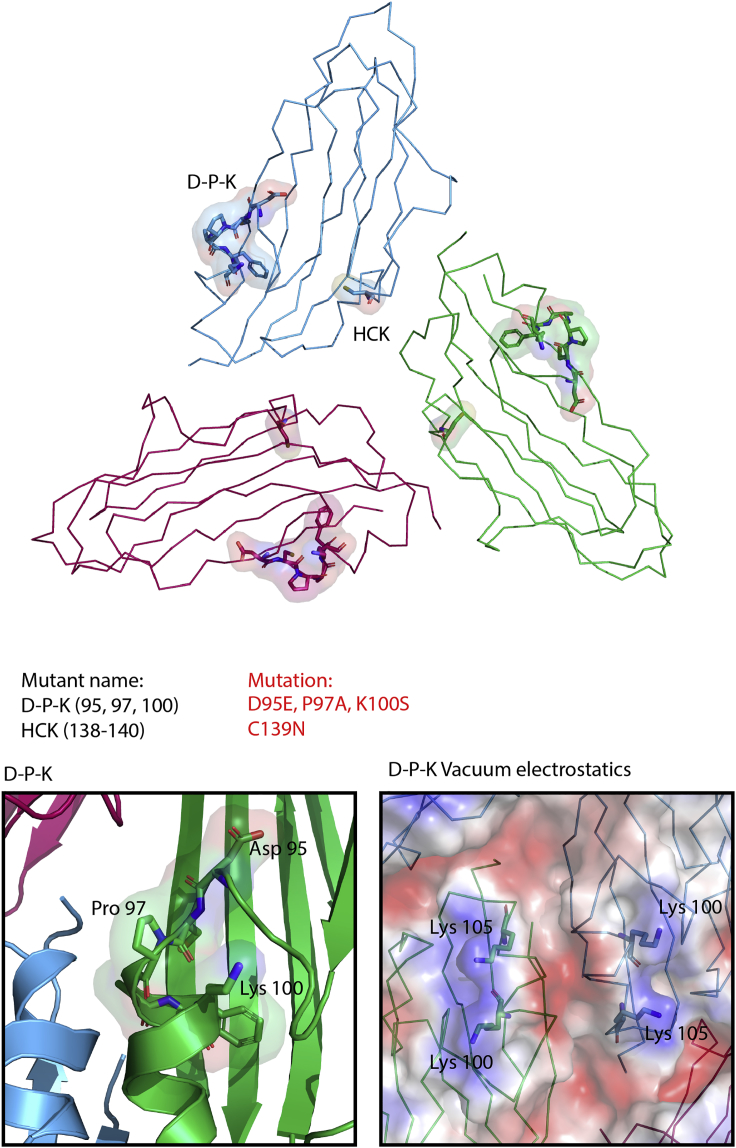
PLRV Surface Mutations Affect Aphid Transmission Efficiency DPK and HCK point mutants from the Kaplan et al. (2007) study had no impact on capsid formation. D95E, P97A, K100S triple mutant decreased aphid transmission efficiency, while C139N did not affect transmission efficiency. PLRV asymmetric unit represented in ribbon form, with mutated residues shown in stick representation. Subunits are colored blue (subunit A), green (subunit B), and red (subunit C). Left panel: zoom view of D95, P97, K100 situated on α1 across the A:B subunit interface. Right panel: vacuum electrostatic representation of DPK motif showing positively charged patch that is disrupted by the K100S mutation (see also Figure S6).

The capsids described here answer long-standing questions about the luteovirids. In the past, insights into their architecture have been limited to those from homology modeling, or on biophysical analyses, such as chemical crosslinking and mass spectrometry (Terradot et al., 2001, Kaplan et al., 2007, Chavez et al., 2012, Doumayrou et al., 2016, Alexander et al., 2017). These studies have helped inform biological hypotheses, but are problematic. One example of this is a PLRV homology model generated by Terradot et al., using a rice yellow mottle virus structure as a template. Despite a relatively high degree of sequence similarity, known sequence epitopes were inaccurately positioned. Residues 83–89, corresponding to epitope 5 in a study by Torrance, were proposed to lie within the βB-βC loop (Torrance, 1992, Terradot et al., 2001), but these residues comprise the entirety of βC in the experimental structure. The new structures presented here allow accurate homology modeling of luteovirid capsids, and we can now present structures from each of the three Luteoviridae genera. A molecular understanding of the mechanisms involved with the transmission of plant viruses via their aphid hosts is much sought after, and the data presented here provide the basis for such studies. For instance, mutations in the CP of PLRV that affect aphid transmission can now be rationalized (see Figures 7 and S6) (Kaplan et al., 2007). The triple mutant D95E, P97A, K100S had no impact upon capsid formation, but decreased transmission efficiency, suggesting that this region of the CP may be important for transmission. Our structures show that D95, P97, and K100 are situated within α1 and the preceding loop, and all three side chains are surface exposed (Figure 7), where they could, for example, affect binding of the virus to receptor(s) in the aphid vector. Much further work is needed, but the structural data presented here and recent identification of luteovirid receptors makes such studies plausible. These structures may also underpin development of rationally designed molecules to control these viruses (Linz et al., 2015, Mulot et al., 2018).

### The VLP Approach to Solving Virus Structures

Plant-based expression of VLPs has been successful in recent years following the development of accessible vector systems for the transient expression of proteins in plants (Sainsbury and Lomonossoff, 2008, Marsian et al., 2017). Such VLPs faithfully replicate the structure and immunogenicity of the wild-type viruses, and so are suitable surrogates for wild-type capsids in structure determination (Buonaguro and Buonaguro, 2014, Hesketh et al., 2015, Hesketh et al., 2017, Marsian et al., 2017). This VLP approach circumvents both the biosafety concerns of working with infectious viruses and difficulties in generating sufficient quantities of viruses that replicate at low titer in their natural hosts, such as non-mechanically transmissible and/or phloem-limited plant pathogens.

Here, however, we demonstrate that VLP expression is not a “magic bullet” that allows all structures to be determined. Transmission of luteovirids by their insect vectors requires that virions cross multiple aphid cell membranes, and completion of the aphid portion of the viral life cycle requires a minor capsid constituent that is not present in our particles. This so-called CP-RTD is encoded by occasional readthrough of the ORF3 stop codon, leading to a ∼54-kDa translation product encoding the CP attached to the RTD by a flexible linker. Such RTDs are common in RNA viruses, but they are almost never observed in structures of even wild-type virus (e.g., bacteriophage Qβ) owing to their low copy number and cryptic mechanism of incorporation into capsids, but primarily owing to the icosahedral symmetry averaging applied in structure determination (Bahner et al., 1990, Filichkin et al., 1994, Golmohammadi et al., 1996). We intentionally excluded CP-RTD from our expression constructs for experimental tractability, but the ability to include minor capsid components or proteins in VLPs presents many interesting questions for future work. Asymmetric structures of VLPs containing variable amounts (and spatial positions) of RTD would be both exceptionally challenging and of enormous interest.

Despite the high degree of similarity across the three type-species structures, we were able to generate monodisperse VLP samples for only two, BYDV and PLRV. PEMV1 VLPs were persistently and irretrievably heterogeneous and were completely intractable for structural characterization by cryo-EM. This may reflect the more complicated wild-type infection setting for PEMV1, which normally exists as a disease complex with the umbravirus PEMV2, a feature absent from both BYDV and PLRV infections. We can speculate that the requirement to encapsidate two different genomic RNAs requires an additional stringency in encapsidation of RNA and/or the protein-protein interactions that drive capsid assembly. This will also be a fascinating question to address in future experiments.

## STAR★Methods

### Key Resources Table

**Table:** 

REAGENT or RESOURCE	SOURCE	IDENTIFIER
**Bacterial and Virus Strains**		

*Agrobacterium tumefaciens* LBA4404	Thermo scientific	18313015
BYDV (source strain for coat protein sequence)	Invitrogen GeneArt	NCBI accession NC_004750
PLRV (source strain for coat protein sequence)	Invitrogen GeneArt	NCBI accession NP_056749
PEMV (source strain for coat protein sequence)	Invitrogen GeneArt	NCBI accession NC_003629.1

**Deposited Data**		

Density map of BYDV VLP	This study	EMDB: EMD-10142
Density map of PLRV VLP	This study	EMDB EMD-10144
Coordinates of BYDV asymmetric unit	This study	PDB: 6SCL
Coordinates of PLRV asymmetric unit	This study	PDB: 6SCO

**Experimental Models**: **Organisms/Strains**		

*Nicotiana benthamiana*	N/A	N/A

**Software and Algorithms**		

RELION 2.1	Kimanius et al., 2016	https://www2.mrc-lmb.cam.ac.uk/relion
MOTIONCOR 2.0	Zheng et al., 2017	http://msg.ucsf.edu/em/software/motioncor2.html
gCTF	Zhang, 2016	https://www.mrc-lmb.cam.ac.uk/kzhang/Gctf/
UCSF Chimera	Schwede et al., 2003, Pettersen et al., 2004	https://www.cgl.ucsf.edu/chimera/ RRID: SCR_004097
Coot	Emsley and Cowtan, 2004	https://www2.mrc-lmb.cam.ac.uk/personal/pemsley/coot/ RRID: SCR_014222
Phenix	Adams et al., 2010	https://www.phenix-online.org/ RRID: SCR_014224

**Other**		

Homology model template for initial model building. *Carnation* mottle *virus coat protein*.	Morgunova et. al. 1994	Protein Data Bank 6SCO

### Lead Contact and Materials Availability

Further information and requests for resources and reagents should be directed to, and will be fulfilled by, the lead contact, Neil A. Ranson (n.a.ranson@leeds.ac.uk).

### Experimental Model and Subject Details

No experimental models were used in this study.

### Method Details

#### Transient Expression of BYDV, PLRV and PEMV VLPs in N. benthamiana

Sequences encoding the CPs of BYDV strain PAV (NC_004750), PLRV NCBI reference strain (NP_056749) and PEMV1 (NC_003629.1) were synthesised by GeneArt. All were codon optimised for *N*. *benthamiana* expression with additional 5’ Kozak sequences and flanked with 5’ and 3’ AgeI and XhoI sites to allow restriction-ligation cloning into pEAQ-*HT*. Sequenced plasmids were transformed into *A*. *tumefaciens* LBA4404, and liquid cultures resuspended in MMA buffer (10mM MES pH 5.6, 10mM MgCl_2_, 100μM Acetosyringone) to an OD600 of 0.4. prior to syringe infiltration into the leaves of 3-week old *N*. *benthamiana* plants. Tissue was harvested 5-8 days after infiltration.

#### BYDV VLP Purification

Harvested tissue was blended in 3x(v/w) phosphate extraction buffer (PEB; 0.1M sodium phosphate buffer pH 7.4, 150mM NaCl) supplemented with cOmplete™ EDTA-free protease inhibitor cocktail (Roche) using a Waring blender. Partial purification was achieved by passing homogenate through miracloth, clarified by centrifugation at 10,000g for 20 minutes, and then layered onto a double sucrose cushion of 0.9mL 70% (w/v) sucrose below 5mL of 25% (w/v) sucrose in PBS. Samples were centrifuged in a SureSpin 630 (Thermo scientific) swing-out rotor at 166,880g for 3 hours at 5°C, and the bottom 1.5mL collected from the 70% and interface region, before dialysis overnight against 5L PEB at 4°C in 100kDa MWCO float-a-lyzers (Spectrum Labs). After dialysis samples were centrifuged at 16,000g for 12 minutes and the supernatant (∼5mL) layered on top of a 10-60% (w/v) Nycodenz gradient consisting 1mL fractions in 10% steps. Centrifugation in a TH-641 (Thermo scientific) swing-out rotor at 273,799g for 3.5 hours at 5°C resulted in an opalescent band which was removed with a needle and syringe and dialysed overnight against PEB.

#### PLRV and PEMV1 VLP Purification

PLRV/PEMV1 CP-expressing tissue was homogenised in 3x (v/w) 0.1M citrate buffer pH 6.0 supplemented with EDTA-free cOmplete™ protease inhibitor tablets using a Waring blender. Blended tissue was clarified by passage through miracloth followed by centrifugation at 20,000g for 20 minutes at 5°C. Partial purification of particles was performed as for BYDV with sucrose solutions dissolved in 0.1M citrate buffer pH 6.0, and the bottom 1.5mL fraction dialysed overnight against 2-5L of citrate buffer.

Following dialysis, samples were centrifuged in a bench-top centrifuge at 16,000g for 12 minutes at 5°C. PLRV VLPs were further purified by layering ∼2mL supernatant on to a 20-50% linear sucrose gradient and centrifuged at 273,799g in a TH-641 swing-out rotor for 2.5 hours at 5°C. 1mL fractions were collected by piercing the bottom of each tube and analysed by SDS-PAGE (Figure S4). Fractions with dominant bands consistent with the expected size of CP were pooled and dialysed against 0.1M citrate overnight. Positive fractions were pooled and dialysed against 5L of citrate buffer at 4°C overnight using 100kDa MWCO float-a-lyzers.

No significant improvements in the morphology of PEMV1 VLPs were seen when changing the salt concentration during extraction and purification (0-500mM NaCl), or by the inclusion of MgCl_2_ or CaCl_2_. Particles could be detected when extracted and purified at pH 6-6.75 in either citrate or MES buffers, however particle integrity was significantly affected at pH 6.75. Purification above this pH, or the use of Nycodenz prevented detectable particle formation. Isopycnic ultracentrifugation of partially purified particles with either CsCl or Optiprep resulted in opalescent bands containing CP but did not yield well-formed particles.

#### Negative Stain Electron Microscopy (nsEM)

nsEM grids were produced by applying 3 μL virus solution to carbon-coated copper grids. Excess liquid was blotted away, the grids were washed in water twice and once in 2% (w/v) uranyl acetate, excess liquid was removed and grids were air-dried. The grids were imaged using either a FEI T-F20 EM, fitted with a FEI CMOS camera or FEI T-12 EM fitted with a Gatan US4000 (Astbury Biostructure Laboratory, University of Leeds).

#### Cryo-EM

Cryo-EM grids were prepared by applying purified VLPs to 400-mesh lacey carbon grids with an additional ultra-thin (<3nm) continuous carbon film (Agar Scientific, UK). Grids were glow-discharged for 30 seconds prior to sample application (easiGlow, Ted Pella). BYDV VLPs were applied to the grids once but for other samples, to increase the number of particles that adhere to the carbon, 3 μl of the sample was incubated on the grid for 5 minutes. The majority of the liquid evaporated during the incubation, but at no point was the grid allowed to dry; this was repeated a number of times (Table S1). The final 3 μl was blotted immediately using FEI vitrobot mark IV (ThermoFisher) device. Grids were vitrified by plunging into in liquid nitrogen-cooled liquid ethane, at 90% relative humidity and 4°C. All data were collected on a ThermoFisher Titan Krios (Astbury Biostructure Laboratory, University of Leeds) EM at 300 kV, using a dose of 63.2 e^-^/Å^2^ for BYDV and 72 e^-^/Å^2^ for PLRV, and a magnification of 75,000x (Table S1). Exposures were recorded using the EPU software on a ThermoFisher Falcon III detector, with an object sampling of 1.065 Å/pixel. Each movie had a total exposure of 1.5 seconds and contained 59 frames. Data collection was set up as described previously (Thompson et al., 2019), and details for each dataset are shown in Table S1.

#### Image Processing

Image processing was carried out using RELION 2.1 (Kimanius et al., 2016). Drift-corrected averages of each movie were created using MOTIONCOR2 (Zheng et al., 2017) and the contrast transfer function of each determined using gCTF (Zhang, 2016). Approximately 1000 particles were manually picked and classified using reference-free 2D classification. The resulting 2D class average views were used for automated particle picking using RELION 2.1 (see Table S1 for details). Automated picking of particles on lacey carbon grids picked on the carbon edge holes (i.e. without particles). To remove ‘junk’ images, the data was further classified using both reference-free 2D classification, and 3D classification with icosahedral symmetry imposed. A 30nm sphere was used for the starting model for PLRV. For BYDV a 60 Å filtered reconstruction of PLRV was used as the starting model. After each round, the best classes/class were taken forward. Post-processing was employed to mask the model, and estimate/correct for the B-factor of the maps. The final resolution was determined using the ‘gold standard’ Fourier shell correlation (FSC = 0.143) criterion (Figure S5). Local resolution was estimated using the local resolution feature in RELION (Figure S5).

#### Model Building and Refinement

For initial model building, a homology model was generated using the SWISS model server and fit into the post processed map of BYDV using UCSF Chimera (Schwede et al., 2003, Pettersen et al., 2004). The template model, selected by the SWISS model server, was the coat protein of carnation mottle virus, pdb code 1OPO (Morgunova et al., 1994). For PLRV, the refined model of BYDV CP, generated in this study, was used as an initial model. Models were inspected with COOT and regions of protein backbone that clearly did not fit the density were deleted (Emsley and Cowtan, 2004). These regions were then built *de novo*. Rotamer fitting and Ramachandran improvement were carried out in COOT. Secondary structure restraints were generated with PHENIX and modified manually where required (Adams et al., 2010). Iterative rounds of model refinement and building/modification were carried out with PHENIX real space refine and COOT respectively. Secondary structure restraints were used where required. Models were validated using MolProbity (Chen et al., 2010).

### Quantification and Statistical Analysis

Structural alignments and principle component analyses were carried out with the DALI web server (http://ekhidna2.biocenter.helsinki.fi/dali/).

### Data and Code Availability

Cryo-EM datasets generated in this study have not been deposited in a public repository but are available from the corresponding author on request. All resulting Cryo-EM maps and models are deposited in the EMDB and PDB respectively. See Key Resources Table.

## Supplemental Information

Document S1. Figures S1–S7 and Tables S1–S5

Document S2. Article plus Supplemental Information
