# Supplementary material for: Combining Transient Expression and Cryo-EM to Obtain High-Resolution Structures of Luteovirid Particles
Source: Structure. 2019 Dec 3;27(12):1761–1770.e3. doi: 10.1016/j.str.2019.09.010 (PMC6899511; doi:10.1016/j.str.2019.09.010)
Supplement: Document S1. Figures S1–S7 and Tables S1–S5 [file mmc1.pdf]

Structure, Volume *27*

Supplemental Information

Combining Transient Expression and Cryo-EM to Obtain High-Resolution Structures of Luteovirid Particles

Matthew J. Byrne, John F.C. Steele, Emma L. Hesketh, Miriam Walden, Rebecca F. Thompson, George P. Lomonossoff, and Neil A. Ranson

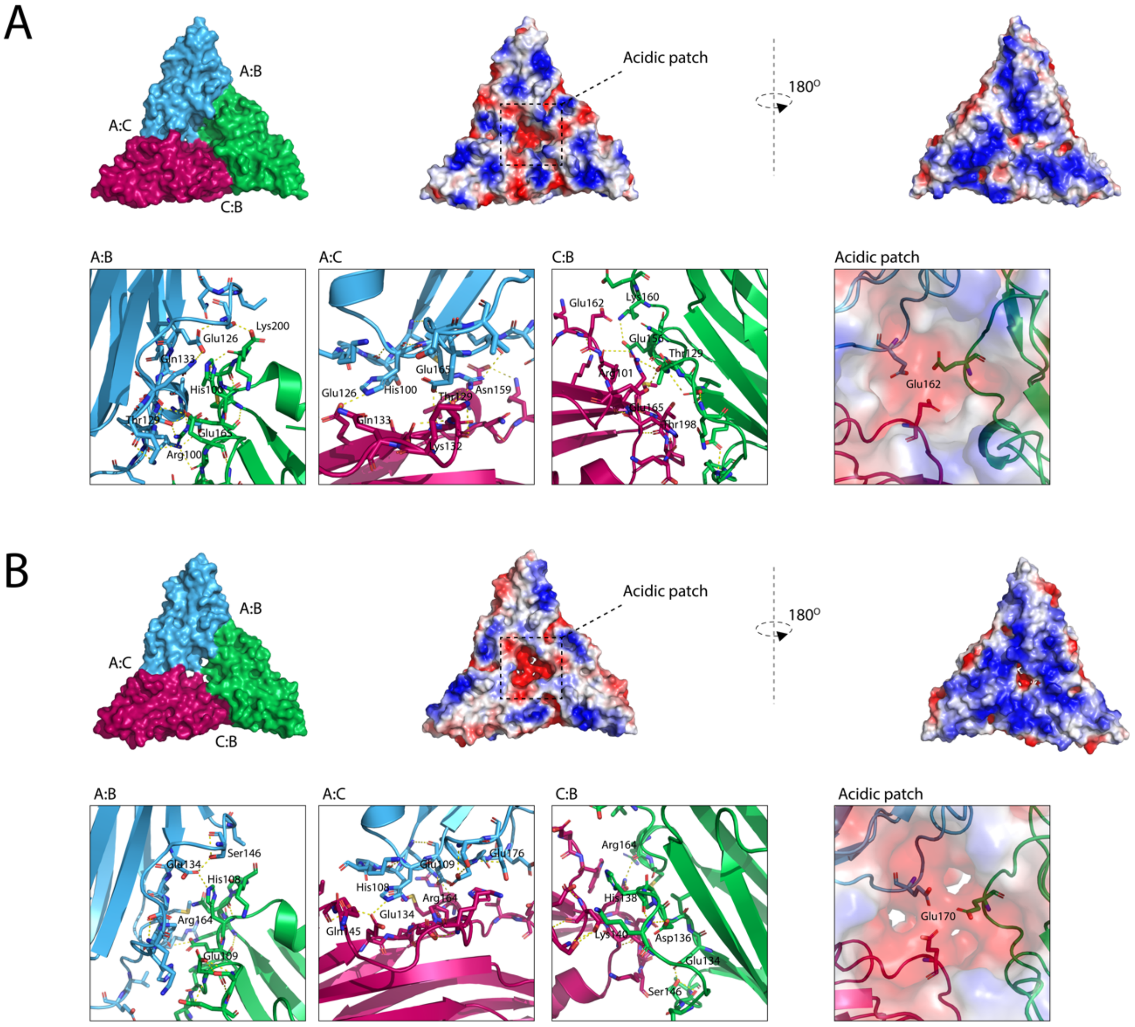

Figure S1. Luteovirid VLP surface charge and inter-subunit interactions (Related to Figure 3). The surface charge of luteovirid capsids proteins is conserved. **Left:** For clarity, CP trimers coloured according to quasi-conformer (A=blue, B=green & C=red), shown from the outer surface of the capsid. **Middle:** Vacuum electrostatic representation of the outer surface of the CP. **Right:** Vacuum electrostatic representation of the inner surface of the CP. **(A)** BYDV **(B)** PLRV. Labelled panels depict interactions at the A:B, A:C and C:B interfaces and central acidic patch. Key residues are labelled for clarity.

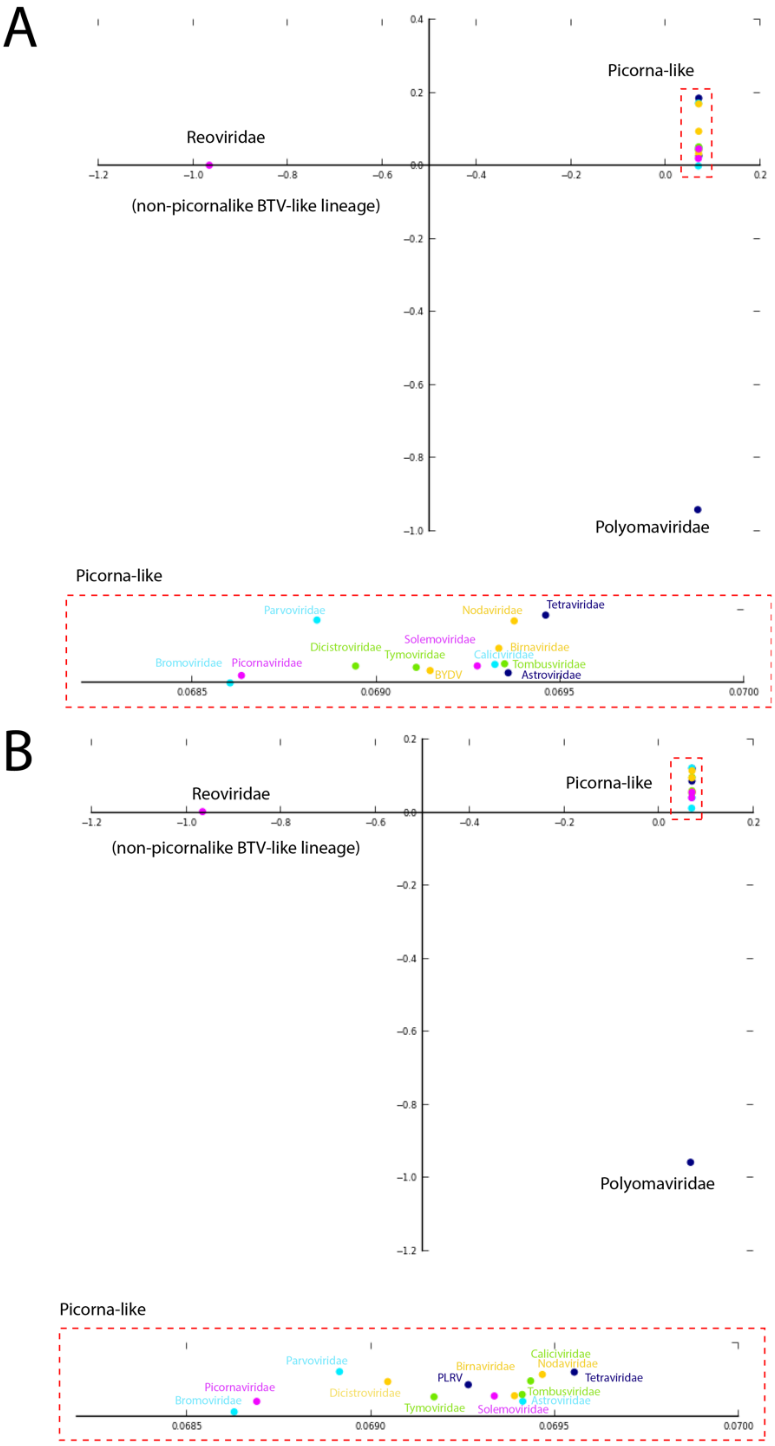

Figure S2. (Related to Figure 4) Correspondence analysis of BYDV/PLRV structural identity against representative members of picorna-like lineage families. A representative member of the structurally distinct BTV-like lineage is included for comparison. Analysis carried out with DALI server. Closely related families are expanded in the red dashed box for clarity. **(A)** BYDV **(B)** PLRV

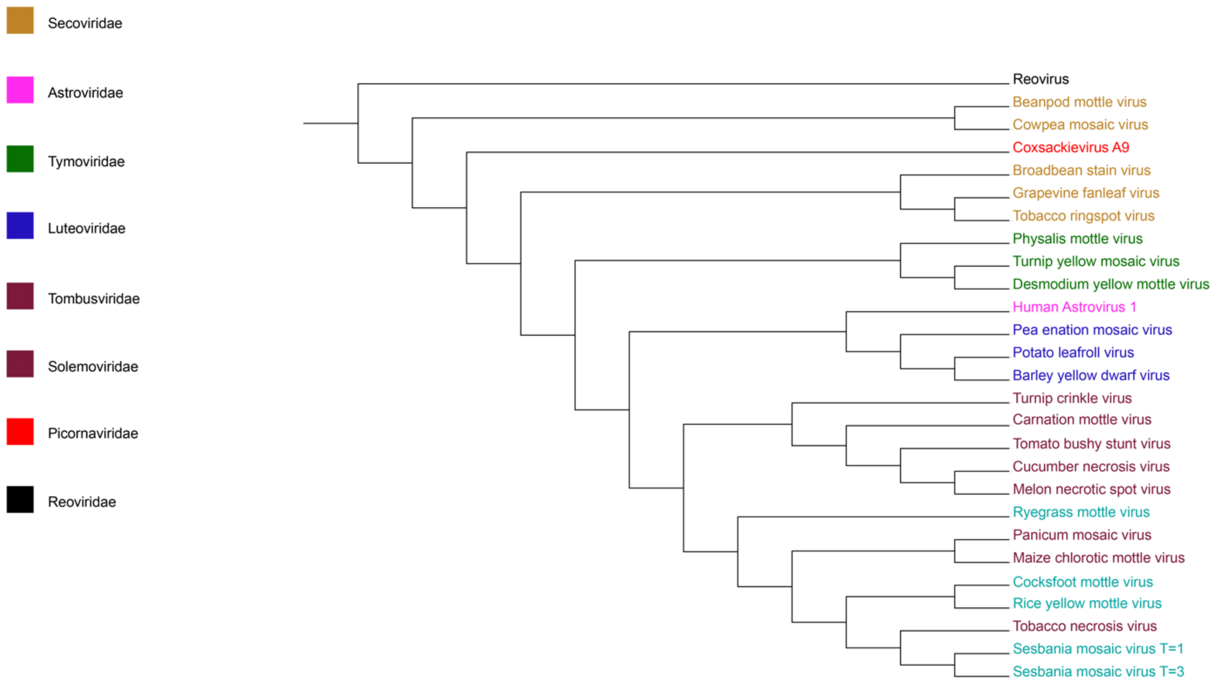

Figure S3. **(Related to Figure 4 and 6) Structural dendrogram demonstrating the Luteoviridae are structurally similar to picorna-like viruses**. Additionally, a picornaviridae member, Coxsackievirus A9, and a non-picorna-like virus, Reovirus, are included for comparison.

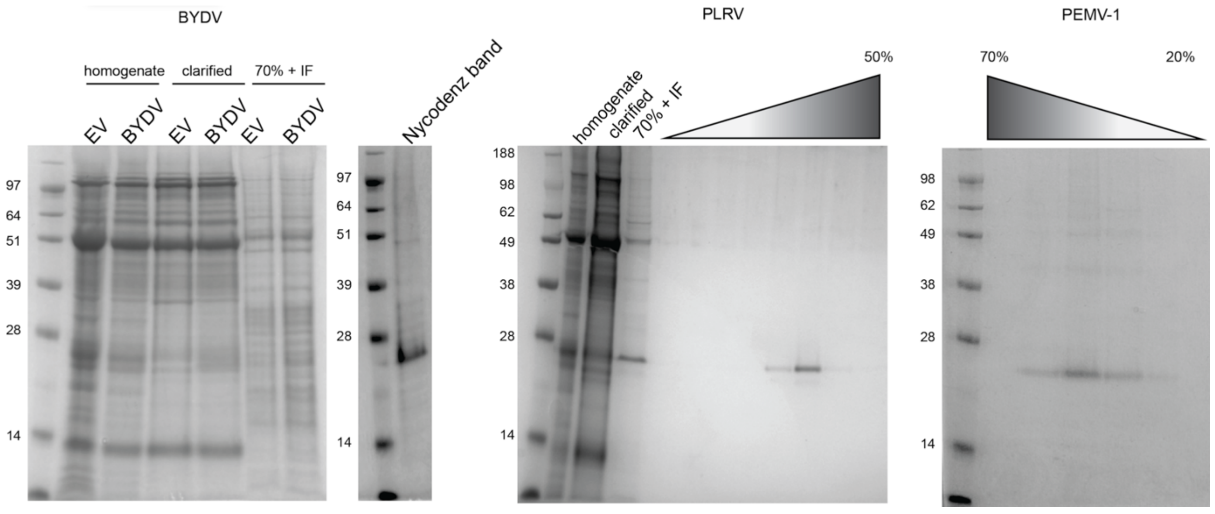

Figure S4. **(Related to figure 2). SDS page analysis of purified Luteovirid VLPs.**

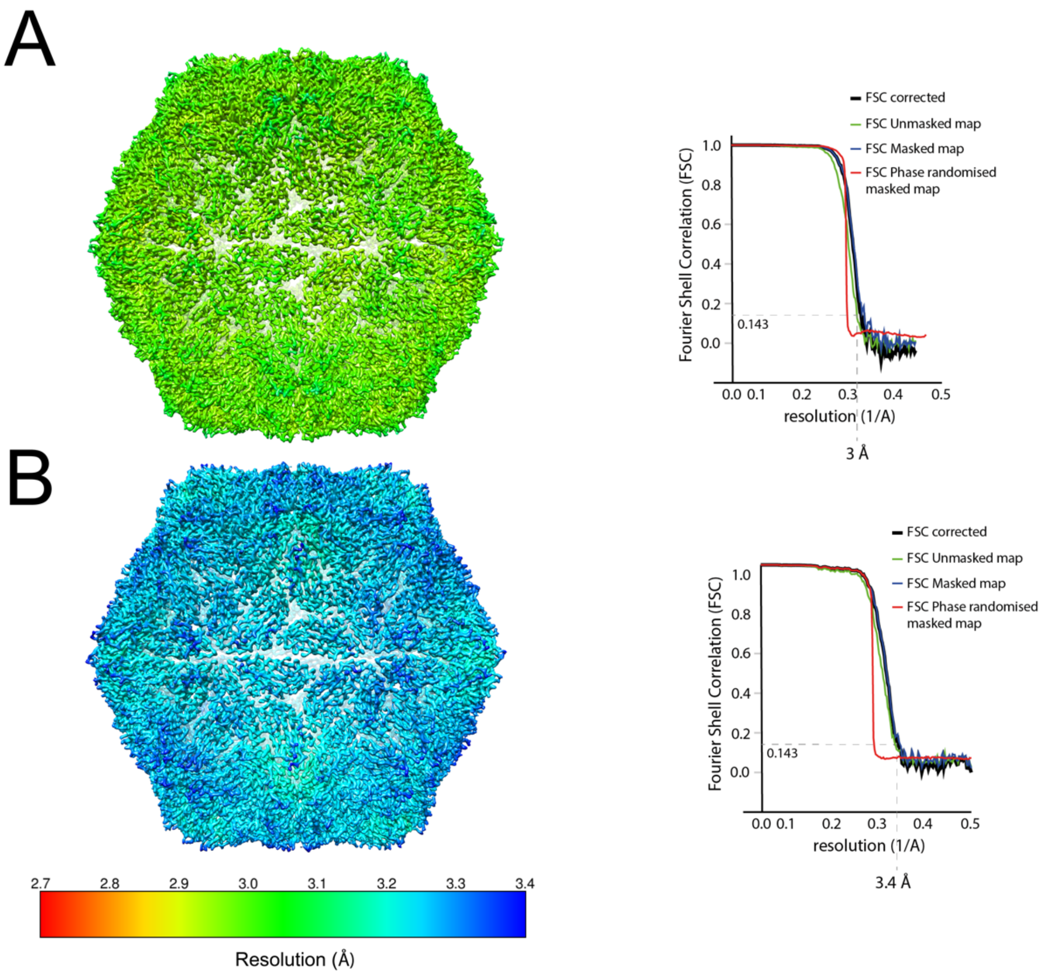

Figure S5. Local resolution and FSC curves for BYDV and PLRV (Related to Figure 3). Left: Local resolution of luteovirid capsid structures. Structures coloured according to the local resolution of the map. **Right:** Fourier shell correlation curves as a function of resolution. The resolution that corresponds to an FSC coefficient of 0.143 was 3.0 and 3.4 for BYDV and PLRV respectively. **(A)** BYDV **(B)** PLRV.

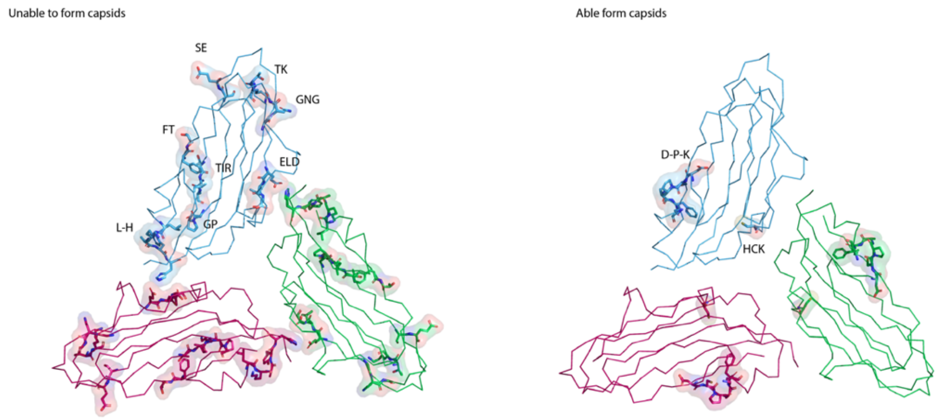

Mutants from Kaplan 2007:

Mutant name:	Mutation:
FT (74,75)	F74A, T75A
GP (90,91)	G90A, P91A
L-H (104, 108)	L104P, H108P
SE (121, 122)	S121A, E122A
ELD (134, 136)	E134A, D136A
GNG (185-187)	G185A, G185A
TIR (199-201)	T199A, R201A
TK (154-155)	T451A, K155A

Mutant name:	Mutation:
D-P-K (95, 97, 100)	D95E, P97A, K100S
HCK (138-140)	C139N

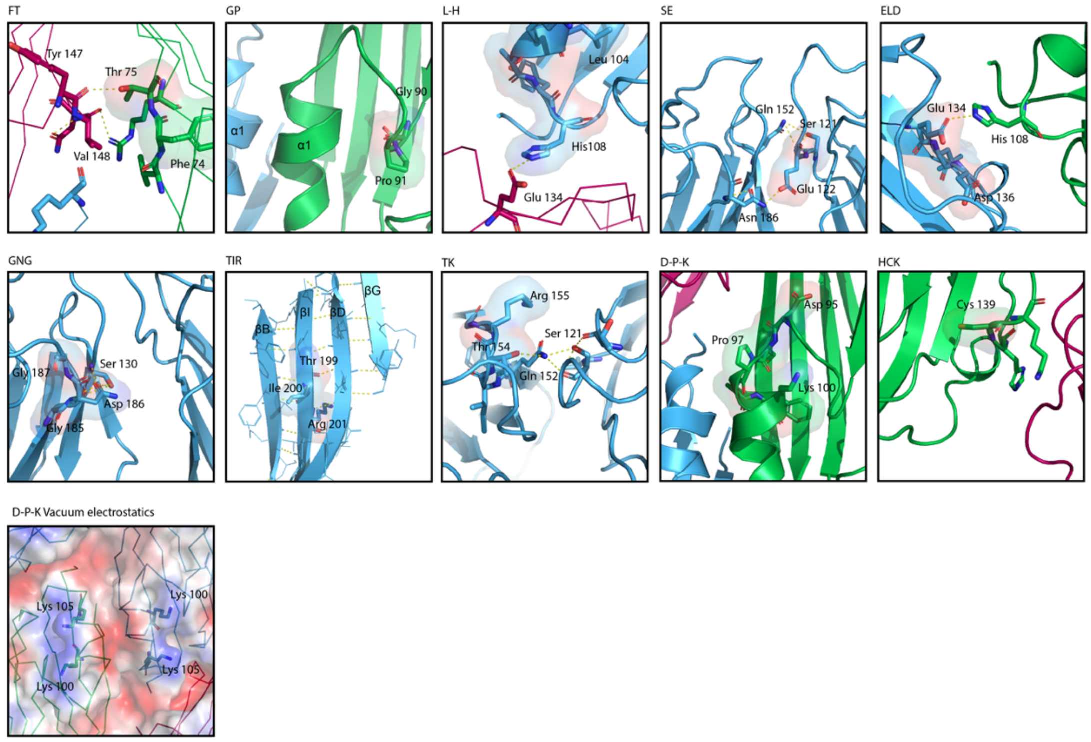

Figure S6. **(Related to Figure 7 and Table S2). PLRV mutant analysis. Top:** Mutations studied in Kaplan 2007 mapped onto experimental structure of PLRV CP from this study. Subunit backbones are shown in ribbon form, mutant regions are shown in stick and space filling representation, coloured according the subunit A (blue), subunit B (green), subunit C (red). Labels indicate mutation (nomenclature as in Kaplan 2007). **Bottom:** Panels showing detailed representations of mutant residues mapped onto the experimental PLRV CP structure.

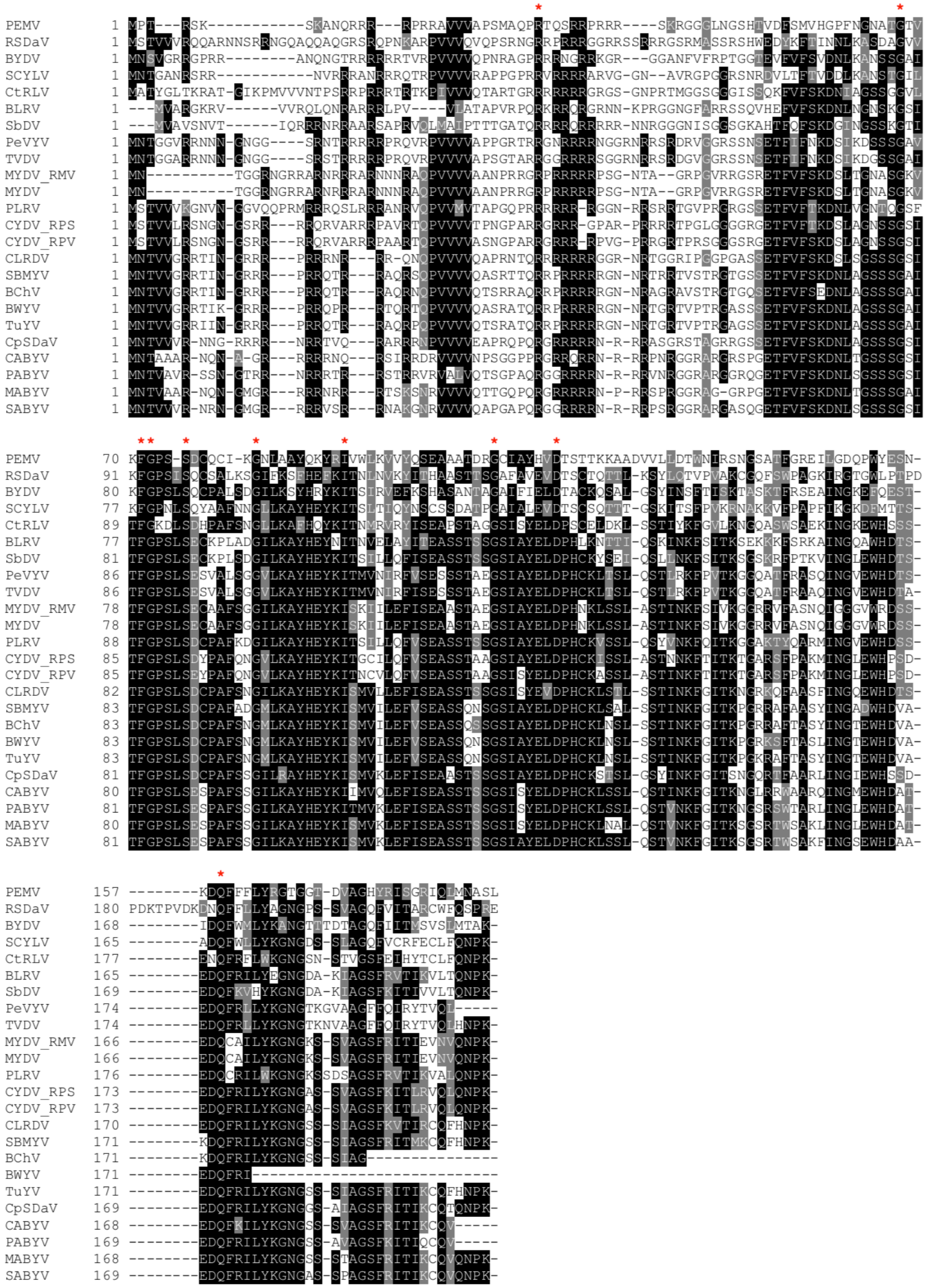

Figure S7. Luteovirid sequence alignment (Related to Figures 4 and 7). Alignment of BYDV, PLRV and PEMV CP sequences with that of other Luteoviridae family members. Conserved residues are indicated by a red asterisk for clarity.

Table S1. (Related to figure 3 and figure 4)

Cryo-EM data collection, refinement and validation statistics.

	PLRV	BYDV
**Data collection and processing**		
Sample applications to grid	3	1
Magnification	75, 000 x	75, 000 x
Voltage (kV)	300	300
Electron exposure (e–/Å^2)	71.65	63.2
Defocus range of micrographs (μm)	-0.2 to -4.5	-0.2 to -4.2
Pixel size (Å)	1.065	1.065
Symmetry imposed	I1	I1
Initial particle images (no.)	91, 498	326, 364
Final particle images (no.)	74, 157	324, 235
Map resolution (Å)	3.4	3.0
FSC threshold	0.143	0.143
Number of frames	59	59
**Refinement**		
Map sharpening *B* factor (Å^2)	-228.3	-198.1
Model composition		
Non-hydrogen atoms	0	0
Protein residues	1087	1070
Nucleic acids	0	0
R.m.s. deviations		
Bond lengths (Å)	0.01	0.01
Bond angles (°)	0.83	0.93
Validation		
Clashscore	2.36	2.02
Poor rotamers (%)	0.00	0.84
Ramachandran plot		
Favored (%)	88.66	94.52
Allowed (%)	11.34	5.48
Disallowed (%)	0.00	0.00

Mutant	Proposed impact
FT	**Thr75:** Loss of H-bond with Tyr147 on neighbouring subunit.<br>**Phe74:** disruption of hydrophobic core of jelly roll.
GP	**Gly90, Pro91:** displacement of α1-βC loop. Disruption of α1-α1 interface between neighbouring subunits.
L-H	**His108:** loss of H-bond with Glu134 of neighbouring subunit.<br>**Leu104:** Disruption of hydrophobic core of jelly roll fold.
SE	**Ser121:** loss of H-bond with Gln152 on neighbouring subunit.<br>**Glu122:** loss of hydrogen bond with Asn186.<br>Disruption of intersubunit interactions.
ELD	**Glu134:** Loss of H-bond with His108 (as in L-H mutant). Disruption of intersubunit interactions.
GNG	**Asn186:** Loss of H-bond with Ser130. Potential displacement of βH-βI and βC-βD loops.
TIR	**Thr199, Ile200, Arg201:** Disruption of BIDG β-sheet interactions.<br>**Thr199, Arg201:** Face to capsid interior, may be involved in encapsidation of RNA.<br>**Ile200:** Disruption of hydrophobic core of jelly roll.
TK	**Thr154:** disruption of H-bond network with Gln152 and Ser121 on neighbouring subunit at 5-fold and β-annulus.
DPK	**Lys100:** Loss of charge at capsid surface, may impact engagement of receptor or other process involved in vector transmission.
HCK	**Cis139:** Sidechain facing jelly roll interior. No apparent impact on capsid formation or transmission.

Table S2. PLRV mutants disrupt capsid formation and transmission efficiency. (Related to Figure 7 and Figure S8). Using the experimentally derived structure of the PLRV capsid from our study, we attempt to rationalise the findings of Kaplan et al. 2007.

	PLRV	BYDV	$PEMV^{BYDV}$	$PEMV^{PLRV}$	$PLRV^{BYDV}$	$BYDV^{PLRV}$
PLRV	-	0.772	0.716	0.100	0.840	1.127
BYDV		-	0.119	0.780	0.074	1.072
$PEMV^{BYDV}$			-	0.829	0.110	0.923
$PEMV^{PLRV}$				-	0.749	1.107
$PLRV^{BYDV}$					-	0.923
$BYDV^{PLRV}$						-

Table S3. (Related to Figure 4) Structural superposition RMSD matrix of all models presented in this study (RMSD in Å).

Analyses of protein:protein interfaces within the AU			
Virus CP	Chain 1	Chain 2	Buried surface area at interface (Å^2)
PLRV	A	B	438.3
PLRV	A	C	457.7
PLRV	B	C	452.6
BYDV	A	B	416.3
BYDV	A	C	406.8
BYDV	B	C	426.6

Table S4. (Related to Figure 3) Analysis of protein:protein interfaces within the asymmetric unit of luteovirid capsid structures. AB:AB interfaces represent the buried surface area between the constituents of AUs opposed around the 5 and 3 fold symmetry axes. BC:BC interfaces represent the buried surface area between the constituents of AUs opposed at the 2-fold symmetry axis.

Analyses of protein:protein interfaces across neighbouring AUs					
Virus CP	Interface AU 1		Interface AU 2		Buried surface area at interface (Å^2)
PLRV	A	B	A	B	1380.2
PLRV	C	B	C	B	1197.9
BYDV	A	B	A	B	1312.2
BYDV	C	B	C	B	1428.7

Table S5. (Related to Figure 3) Analysis of protein:protein interfaces across neighbouring asymmetric units in luteovirid capsid structures. AB:AB interfaces represent the buried surface area between the constituents of AUs opposed around the 5 and 3 fold symmetry axes. BC:BC interfaces represent the buried surface area between the constituents of AUs opposed at the 2-fold symmetry axis.
